# Utility of Diagnostic Arthroscopy Before Medial Patellofemoral Ligament Reconstruction in Pediatric and Adolescent Patients Without Articular Findings on Magnetic Resonance Imaging

**DOI:** 10.1177/23259671261430753

**Published:** 2026-04-07

**Authors:** Holden Archer, Kaleb Patterson, Jonathan D. Schwartzman, Scott D. McKay, Indranil Kushare

**Affiliations:** † Pediatric Orthopaedic Surgery, Baylor College of Medicine, Houston, Texas, USA; Investigation performed at Baylor College of Medicine, Houston, Texas, USA

**Keywords:** medial patellofemoral ligament, diagnostic arthroscopy, pediatric knee, medial patellofemoral ligament reconstruction

## Abstract

**Background:**

Medial patellofemoral ligament (MPFL) reconstruction is frequently used to manage recurrent patellar instability. Diagnostic arthroscopy is commonly performed before MPFL reconstruction to identify, confirm, and address intra-articular pathology. However, when preoperative magnetic resonance imaging (MRI) does not demonstrate intra-articular lesions, it is unclear how often diagnostic arthroscopy in pediatric patients alters surgical management.

**Purpose:**

To determine the utility of diagnostic arthroscopy in young patients undergoing MPFL reconstruction who had no intra-articular chondral or osteochondral pathology, meniscal tears, or loose bodies identified on preoperative MRI, and to characterize intra-articular pathology detected on arthroscopy but not identified on MRI.

**Study Design:**

Case series; Level of evidence, 4.

**Methods:**

This was a retrospective cohort study of patients who underwent MPFL reconstruction from 2014 to 2025. Inclusion criteria were age ≤18 years at the time of surgery, available preoperative MRI without intra-articular chondral or osteochondral pathology, meniscal tears, or loose bodies; and MPFL reconstruction with concomitant diagnostic arthroscopy. Exclusion criteria were congenital or syndromic patellar instability, prior ipsilateral knee surgery, absence of diagnostic arthroscopy, and MRI with intra-articular cartilage damage, osteochondral fractures, subchondral fractures, loose bodies, or meniscal tears.

**Results:**

A total of 626 knees were initially identified. After application of exclusion criteria, the final cohort included 81 knees. The mean age was 14.9 years, and 77% patients were female. Of the knees excluded for operative MRI findings, the most common were loose bodies, articular cartilage injuries, and osteochondral fractures. In the final cohort, 35 of 81 (43.2%) knees had arthroscopic pathology, which included chondral changes in multiple sites (n = 35; 43%), loose bodies (n = 3; 4%), and meniscal tears (n = 1; 1%). Overall, 10 of 81 (12.3%) knees underwent changes in management based on arthroscopic findings, including chondroplasty (n = 8; 10%) and loose-body removal (n = 3; 4%).

**Conclusion:**

Diagnostic arthroscopy altered management in 12.3% of pediatric MPFL reconstructions in the absence of intra-articular chondral or osteochondral pathology, meniscal tears, or loose bodies on MRI. These results suggest that routine diagnostic arthroscopy may provide clinically meaningful value only in a subset of patients by identifying pathology not appreciated on MRI.

Patellar instability is a common disorder characterized by dislocation or subluxation of the patella, almost always laterally, relative to the femoral trochlea.^10,19^ Re-dislocation is frequent after initial dislocation, with up to 54% of pediatric patients developing recurrent instability.^8,10,18,19,24^ The peak incidence occurs between 15 and 19 years of age, with an annual incidence of 11.19 per 100,000 person-years.^10,16^ Addressing patellar instability is important because it may lead to substantial functional limitations and early osteoarthritis.^1,2,13^

The medial patellofemoral ligament (MPFL) is the primary soft tissue restraint to lateral patellar displacement,^3,11^ and MPFL reconstruction has been shown to yield excellent functional outcomes.^6,17,24^ Diagnostic arthroscopy at the time of patellar realignment surgery has been reported to identify pathology that would otherwise be missed.^14^ Magnetic resonance imaging (MRI) is routinely used to evaluate the MPFL and to detect intra-articular knee pathology.^7,15,24^

A diagnostic arthroscopy is commonly performed before MPFL reconstruction to assess and manage any intra-articular pathology. However, in the absence of intra-articular chondral or osteochondral pathology, meniscal tears, or loose bodies on preoperative MRI, the extent to which diagnostic arthroscopy alters management in pediatric patients is unclear. Similar investigations, although limited, have primarily examined adult cohorts rather than pediatric patients.^21^ Given differences in anatomy and skeletal immaturity, findings in the pediatric population may not be directly comparable.^9,23,26^

Our primary objective was to determine, among patients ≤18 years undergoing MPFL reconstruction with diagnostic arthroscopy, how often arthroscopy altered management when preoperative MRI demonstrated no intra-articular chondral or osteochondral pathology, meniscal tears, or loose bodies. Our secondary objective was to characterize intra-articular pathology identified on diagnostic arthroscopy but not detected on preoperative MRI. We hypothesized that pediatric patients without intra-articular chondral or osteochondral pathology, meniscal tears, or loose bodies on MRI would rarely require additional procedures at the time of diagnostic arthroscopy.

## Methods

### Study Design and Population

This was a single-center retrospective cohort study involving 5 surgeons (S.D.M., I.K.) and was approved by our institutional review board. We queried our quaternary care center's institutional database for all patients who underwent a Current Procedural Terminology (CPT) code 27427 procedure from 2014 to 2025. After constructing this initial cohort, we applied inclusion and exclusion criteria.

Inclusion criteria were age ≤18 years at the time of surgery, MPFL reconstruction with concomitant diagnostic arthroscopy, and available preoperative MRI without intra-articular chondral or osteochondral pathology, meniscal tears, or loose bodies. Exclusion criteria were age >18 years, unavailable MRI reports from both the radiologist and treating orthopaedic surgeon, congenital or syndromic patellar instability, prior ipsilateral knee surgery, absence of diagnostic arthroscopy, and intra-articular MRI findings including articular cartilage damage, osteochondral fractures, subchondral fractures, loose bodies, or meniscal tears. This study was designed and reported in accordance with STROBE (Strengthening the Reporting of Observational Studies in Epidemiology) guidelines.

### Data Collection

We collected patient and clinical characteristics from the electronic medical record, including age at time of surgery, knee laterality, sex, race, and body mass index (BMI). From the most recent MRI report before surgery, we recorded the date and presence of any reported intra-articular chondral or osteochondral pathology, meniscal tears, or loose bodies. At our institution, all MRI scans are interpreted by fellowship-trained (musculoskeletal [MSK]) radiologists. For surgical procedures, we recorded the date, all procedures performed, arthroscopic findings, and any arthroscopic interventions.

### Data Analysis

Descriptive statistics were used to summarize patient characteristics, MRI findings, arthroscopic findings, and surgical interventions. Categorical variables are summarized as counts and percentages, and quantitative variables as means with standard deviations. The primary outcome was the proportion of cases in which diagnostic arthroscopy altered management when preoperative MRI demonstrated no significant intra-articular pathology. Since no arthroscopic procedures were planned in advance, diagnostic arthroscopy was considered to have altered management when at least 1 additional procedure was performed based on findings at the time of diagnostic arthroscopy. Procedures performed solely based on examination under anesthesia, such as lateral release, were not classified as management changes attributable to diagnostic arthroscopy. The secondary outcome was the frequency and types of intra-articular pathologies identified on diagnostic arthroscopy but not detected on preoperative MRI.

## Results

**Figure 1.**
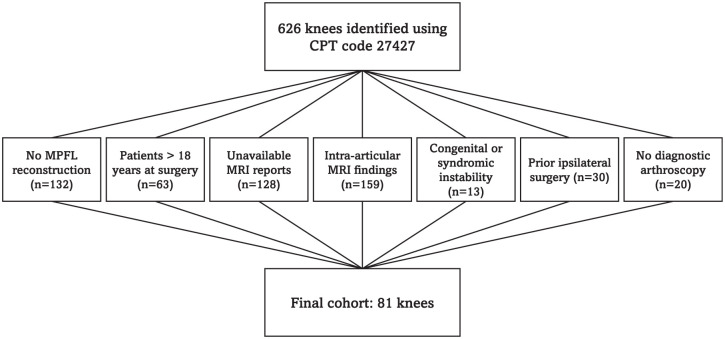
Cohort construction. Knees meeting any exclusion criteria were removed. CPT, Current Procedural Terminology; MPFL, medial patellofemoral ligament; MRI, magnetic resonance imaging.

A total of 626 knees were identified using CPT code 27427. After excluding cases without MPFL reconstruction (n = 132); patients >18 years of age at surgery (n = 63); cases with unavailable MRI reports (n = 128); those with MRI findings of intra-articular chondral or osteochondral pathology, meniscal tears, or loose bodies (n = 159); cases with congenital or syndromic instability (n = 13); those with prior ipsilateral surgery (n = 30), and cases with an absence of diagnostic arthroscopy (n = 20), the final cohort included 81 knees from 77 patients (Figure 1).

### Patient Characteristics

Of the 81 patients, 62 (76.5%) were female. The mean age was 14.9 years (SD, 2.0 years). The mean BMI was 26.2 kg/m^2^ (SD, 6.7 kg/m^2^). The mean time from MRI to surgery was 110.7 days (SD, 120.5 days). Patient characteristics are summarized in Table 1.

**Table 1:** Patient and Clinical Characteristics*^a^*

	Value
Sex	
Female	62 (76.5)
Male	19 (23.5)
Race	
White	60 (74.1)
Black or African American	8 (9.9)
Asian	3 (3.7)
≥2 races	2 (2.5)
Native Hawaiian or Other Pacific Islander	1 (1.2)
Unknown	7 (8.6)
Laterality	
Right	45 (55.6)
Left	36 (44.4)
Age, y	14.9 (2.0)
Body mass index, kg/m^2^	26.2 (6.7)
Time between MRI and surgery, days	110.7 (120.5)

a Data are presented as n (%) or mean (SD). MRI, magnetic resonance imaging.

### Exclusions Based on MRI Findings

Of the 159 knees excluded for relevant intra-articular MRI findings, the most common pathologies were loose bodies (n = 101; 63.5%), lateral femoral condyle articular cartilage injuries (n = 58; 36.5%), femoral osteochondral fracture (n = 54; 34.0%), and patellar osteochondral fractures (n = 53; 33.3%). There were 42 (26.4%) patellar articular cartilage injuries, 12 (7.5%) subchondral fractures, 10 (6.3%) lateral meniscus tears, and 8 (5.0%) medial meniscus tears. There were 5 (3.1%) tibial osteochondral fractures and 1 (0.6%) lateral tibial plateau articular cartilage injury. The MRI findings leading to exclusion are summarized in Table 2.

**Table 2:** MRI Findings Leading to Exclusion*^a^*

	No. (%)
Loose body	101 (63.5)
Lateral femoral condyle articular cartilage injury	58 (36.5)
Osteochondral fracture (femur)	54 (34.0)
Osteochondral fracture (patella)	53 (33.3)
Patellar articular cartilage injury	42 (26.4)
Subchondral fracture	12 (7.5)
Lateral meniscus tear	10 (6.3)
Medial meniscus tear	8 (5.0)
Osteochondral fracture (tibia)	5 (3.1)
Lateral tibial plateau articular cartilage injury	1 (0.6)

a Counts exceed 159 because some knees had >1 relevant MRI finding. MRI, magnetic resonance imaging.

### Surgical Procedures

All patients underwent MPFL reconstruction. The most common additional procedure was tibial tubercle osteotomy (n = 33; 40.7%), followed by femoral osteotomy (n = 9; 11.1%), open lateral release (n = 3; 3.7%), hemi-epiphysiodesis (n = 2; 2.5%), and partial patellectomy (n = 1; 1.2%). The open procedures performed are summarized in Table 3.

**Table 3:** Surgical Procedures Performed

	No. (%)
Medial patellofemoral ligament reconstruction	81 (100)
Tibial tubercle osteotomy	33 (40.7)
Femoral osteotomies (lateral opening wedge, shaft, de-rotation)	9 (11.1)
Open lateral release	3 (3.7)
Hemi-epiphyseal arrest	2 (2.5)
Partial patellectomy	1 (1.2)

### Arthroscopic Pathology

In the final cohort, 35 (43.2%) knees had arthroscopic pathology compared with 46 (56.8%) knees that did not. The most common abnormalities were chondral changes, which were found in 35 (43.2%) knees. Less common findings included loose bodies (n = 3; 3.7%) and medial meniscus tears (n = 1; 1.2%). Arthroscopic findings are summarized in Table 4.

**Table 4:** Arthroscopic Pathology Identified*^a^*

	No. (%)
No pathology identified	46 (56.8)
Chondral changes (any site)	35 (43.2)
Loose body	3 (3.7)
Medial meniscus tear	1 (1.2)

a Counts exceed 81 because some knees had >1 arthroscopic finding.

### Arthroscopic Procedures

No arthroscopic intervention was performed in 55 (67.9%) knees. The remaining 26 (32.1%) knees underwent at least 1 arthroscopic procedure. In 16 of these cases, the only intervention was lateral release, which was performed based on examination under anesthesia rather than arthroscopic findings and therefore was not considered as a management change based on diagnostic arthroscopy. Excluding these cases, 10 knees (12.3% of the total cohort) had management changes directly attributable to diagnostic arthroscopy. These included chondroplasty (n = 8) and loose-body removal (n = 3). In total, 20 (24.7%) arthroscopic lateral releases were performed, of which 4 were in conjunction with another arthroscopic procedure. Arthroscopic procedures are detailed in Table 5.

**Table 5:** Arthroscopic Procedures Performed*^a^*

	No. (%)
No arthroscopic procedures	55 (67.9)
Lateral release	20 (24.7)
Chondroplasty	8 (9.9)
Loose-body removal	3 (3.7)

a Counts exceed 81 because some knees had multiple arthroscopic procedures.

## Discussion

There is limited literature assessing the utility of diagnostic arthroscopy at the time of MPFL reconstruction for identifying intra-articular pathology not apparent on MRI. Because diagnostic arthroscopy increases cost, operative time, and resource use, it is important to critically evaluate whether it provides meaningful benefit in this clinical context. To our knowledge, no prior studies have specifically examined this question in the pediatric population. Our hypothesis was partially supported: while most patients without significant MRI findings did not require additional arthroscopic intervention, 12.3% had pathology identified intraoperatively that altered management. Moreover, arthroscopic lateral release was commonly performed. Although these cases did not represent management changes driven by diagnostic arthroscopy, they demonstrate that an arthroscopic intervention was still often necessary regardless of intraoperative visualization.

### Changes in Management

In our cohort, diagnostic arthroscopy led to changes in 10 (12.3%) knees, representing a subset of cases. The most common additional procedure was chondroplasty (9.9%). In comparison, Shultz et al^21^ reported additional arthroscopic procedures in 31.7% of their adult cohort without planned intra-articular intervention. The majority of additional interventions in their series were partial meniscectomies (22%), whereas our cohort had none. Chondroplasty rates were similar (9.9% vs 7.3%) as were rates of loose-body removals (3.7% vs 2.4%). The absence of meniscectomies in our cohort may reflect differences in patient populations (our cohort was younger with a mean age of 14.9 vs 23 years), and meniscal tears are less common in skeletally immature patients.^9,26^ Additionally, it is also possible that meniscal pathology was more readily detected on MRI in our study, resulting in exclusion from our cohort.

The role of arthroscopic lateral release deserves mention. Although 26 (32.1%) knees underwent an arthroscopic procedure, most of these were lateral releases performed based on examination under anesthesia rather than arthroscopic findings. Excluding these, only 10 (12.3%) knees had management changes directly attributable to diagnostic arthroscopy. Nevertheless, lateral release still represented a frequent additional procedure, highlighting that arthroscopy may add limited but meaningful value in this setting. Importantly, although lateral release was not driven by arthroscopic visualization, it still required arthroscopic access, underscoring that the capability for arthroscopy remains necessary despite a relatively low rate of arthroscopy-driven management changes.

Among the 3 cases in which loose bodies were identified and removed intraoperatively despite a negative MRI result, 1 case had a prolonged MRI-to-surgery interval of 156 days with documented interval instability, which may explain the discrepancy. The other 2 cases had shorter MRI-to-surgery intervals (20-60 days) and documented histories of recurrent instability, although the records did not explicitly note instability episodes within the MRI-to-surgery interval.

Among the 8 knees that underwent chondroplasty for chondral pathology not identified on MRI, the MRI-to-surgery interval ranged from 8 to 371 days with a mean of 111 days, consistent with the overall cohort mean. Four cases had at least 1 documented episode of instability during this interval (48, 156, 194, and 371 days), while the remaining 4 cases (8, 20, 37, and 52 days) had no such episodes documented in the medical record. This heterogeneity suggests that the discrepancy between MRI and arthroscopic findings is not fully explained by the MRI-to-surgery interval or by interval instability events alone.

### Arthroscopic Findings

Discordance between MRI and arthroscopy has been previously documented.^15,21^ Our focus was to identify which intra-articular pathologies were missed by MRI. Luhmann et al^15^ reported MRI-arthroscopy agreement for meniscal tears (kappa = 0.71-0.76) and osteochondral fractures (kappa = 0.65), consistent with moderate MRI performance in detecting these pathologies in the pediatric population. However, Luhmann et al did not examine chondral changes, which were the most common findings in our study. Additionally, their limited sample size (such as only 5 patients with osteochondral fracture) restricts conclusions, and small interpretative differences among radiologists may have influenced results.

Adult studies report adequate sensitivity and specificity of MRI in detecting cartilage pathology, although no sequence is fully reliable.^5,20,25^ In contrast, in the pediatric population, some studies have questioned the utility of MRI in the evaluation of intra-articular knee pathology.^12,22^ This may explain why MRI underestimated certain degenerative changes such as chondral changes. The relatively low rate of missed pathology overall in our study compared to prior literature may also reflect the use of fellowship-trained MSK radiologists at our institution, as well as continued advances in MRI technology that improve diagnostic sensitivity.^4^

### Limitations

There are important limitations to consider. This was a single-center retrospective study, limiting generalizability despite the involvement of multiple surgeons. Similarly, the final sample of 81 knees restricts generalizability. Approximately one-fifth of knees were excluded due to unavailable MRI reports, raising the possibility of selection bias. Retrospective reliance on operative notes and MRI reports introduces variability, as thresholds for reporting or treating pathology may differ among surgeons and radiologists. Additionally, the decision to perform arthroscopic lateral release was based on physical examination under anesthesia rather than diagnostic arthroscopy, and thresholds for this procedure may vary across surgeons. Importantly, we did not assess clinical outcomes, so the impact of treating arthroscopy findings on pain, function, or other results is not known. While prior adult studies have suggested that diagnostic arthroscopy may not change postoperative pain scores,^21^ differences in patient populations and pathology profiles mean these conclusions cannot be directly applied to our cohort. The mean interval between MRI and surgery was 111 days, which may have allowed for additional intra-articular damage to occur after the MRI and before arthroscopy; however, this time frame reflects real-world clinical practice. We acknowledge that the utility of diagnostic arthroscopy may extend beyond intraoperative decision-making; arthroscopy can sometimes provide information regarding trochlear morphology and cartilage status that is not fully captured by imaging or reflected by a change in surgical management and may offer insight into mechanisms of recurrent instability in select patients.

## Conclusion

Diagnostic arthroscopy altered management in 12.3% of pediatric MPFL reconstructions in the absence of intra-articular chondral or osteochondral pathology, meniscal tears, or loose bodies on MRI. These results suggest that routine diagnostic arthroscopy may provide clinically meaningful value in a subset of patients by identifying pathology not appreciated on MRI.

Future work should aim to identify which pediatric patients without significant MRI findings may still benefit from diagnostic arthroscopy. Prospective studies including cost and outcome analyses would help refine patient selection criteria and better define the role of routine diagnostic arthroscopy in this setting.
